# Supplementary material for: Body Weight Fluctuation as a Risk Factor for Type 2 Diabetes: Results from a Nationwide Cohort Study
Source: J Clin Med. 2019 Jun 30;8(7):950. doi: 10.3390/jcm8070950 (PMC6678837; doi:10.3390/jcm8070950)
Supplement: Supplementary file 1 [file jcm-08-00950-s001.pdf]

Supplementary Table S1. Risk of new-onset diabetes according to different indices of body weight variability

Variability	Group	N	Incident diabetes	Duration	Incidence[a]	Model 1[b]	Model 2[c]	Model 3[d]
SD	Q1	889880	16021	3884226.17	4.12	1.00	1.00	1.00
	Q2	1035817	19209	4513638.17	4.26	1.03 (1.00,1.05)	1.01 (0.99,1.03)	0.99 (0.97,1.01)
	Q3	972232	19209	4228263.25	4.54	1.17 (1.15,1.20)	1.11 (1.09,1.14)	1.00 (0.98,1.02)
	Q4	957955	19936	4156898.68	4.80	1.46 (1.43,1.49)	1.34 (1.31,1.36)	1.1(1.08,1.12)
CV	Q1	963334	20072	4194664.27	4.79	1.00	1.00	1.00
	Q2	964225	18669	4200934.1	4.44	0.96 (0.94,0.98)	0.97 (0.95,0.99)	0.99 (0.97,1.01)
	Q3	963652	17790	4196156.43	4.24	0.96 (0.94,0.98)	0.98 (0.96,0.99)	0.99 (0.97,1.01)
	Q4	964673	17844	4191271.47	4.26	1.07 (1.05,1.09)	1.08 (1.06,1.10)	1.08 (1.06,1.1)
VIM	Q1	965888	20014	4205958.2	4.76	1.00	1.00	1.00
	Q2	961433	18517	4188948.7	4.42	0.97 (0.95 ,0.99)	0.97 (0.95,0.99)	0.98 (0.96,1.00)
	Q3	965242	17922	4203140.5	4.26	0.98 (0.96,1.00)	0.98 (0.96,1.00)	0.99 (0.97,1.01)
	Q4	963321	17922	4184978.87	4.28	1.09 (1.06,1.11)	1.09 (1.07,1.12)	1.08 (1.05,1.10)

[a]Incidence rate per 1000 person-years
[b]Model 1 was non-adjusted
[c]Model 2 was adjusted for age, sex, smoking status, alcohol consumption, physical activity, income, hypertension, dyslipidemia and fasting plasma glucose
[d]Model 3 was adjusted for the variables in model 2 plus baseline BMI
Q, quartiles; SD, standard deviation; CV, coefficient of variation; VIM, variability independent of mean
